# Risk of Cardiovascular Events and Medical Cost of Dapagliflozin and Dipeptidyl Peptidase-4 Inhibitors

**DOI:** 10.3389/fphar.2021.689885

**Published:** 2021-09-28

**Authors:** Jong-Mi Seong, Jong Joo Kim, Hae Jin Kim, Hyun Soon Sohn

**Affiliations:** ^1^ Research Institute for Pharmaceutical Sciences, Ewha Womans University, Seoul, South Korea; ^2^ Pharmaceutical Information Research Institute, CHA University, Seongnam, South Korea; ^3^ Department of Endocrinology and Metabolism, Ajou University School of Medicine, Suwon, South Korea; ^4^ College of Pharmacy, CHA University, Pocheon-si, South Korea

**Keywords:** diabetes mellitus, dapagliflozin, dipeptidyl peptidase-4 inhibitor, major adverse cardiovascular event, direct medical cost

## Abstract

This study compared dapagliflozin, a sodium-glucose co-transporter 2 inhibitor, and dipeptidyl peptidase-4 inhibitors (DPP-4i) with regard to cardiovascular (CV) event incidence and direct medical costs during type 2 diabetes treatment. A retrospective cohort study was conducted using national health insurance claims data from September 1, 2014, to June 30, 2018, of patients in Korea. Patients who were prescribed dapagliflozin and DPP-4i for the first time were included. The primary outcome was the incidence of a composite of major adverse CV events (MACEs)—nonfatal myocardial infarction, nonfatal stroke, or in-hospital CV death. Proportional hazard models after propensity score weighting were used to determine hazard ratios (HRs) and 95% confidence intervals (CIs) for MACE in the dapagliflozin and DPP-4i groups. A decision analytic model was used to compare direct medical costs between the two treatment groups from a healthcare provider’s perspective. Of the 260,336 patients in the cohort, 23,147 and 237,189 received dapagliflozin and DPP-4i, respectively. During the follow-up, 184 patients receiving dapagliflozin and 3,674 receiving DPP-4i (incidence, 6.47 and 11.33 events/1,000 person-years, respectively) had MACE. The adjusted HR of MACE for dapagliflozin compared with that for DPP-4i was 0.69 (95% CI 0.57–0.83). The corresponding HRs were consistent among patients with and without underlying CV disease. The estimated direct medical cost appeared to be lower by $68,452 in the dapagliflozin group than that in the DPP-4i group for 3 years, in 1,000 hypothetical patients. In this population-based cohort study, the use of dapagliflozin instead of DPP-4i was associated with a reduced risk of MACE, which subsequently reduced direct medical costs. These data provide valuable information to patients, practitioners, and authorities regarding the risk of CV events associated with dapagliflozin versus DPP-4i use in clinical practice.

## Introduction

Patients with diabetes have an increased risk of developing cardiovascular disease (CVD), the leading cause of mortality in patients with diabetes (Fox et al., 2004; Fox et al., 2007; Franco et al., 2007). Therefore, it is important to characterize the effectiveness of diabetes therapies in reducing the CVD risk.

Dapagliflozin is a sodium-glucose co-transporter 2 inhibitor (SGLT2i) that blocks glucose resorption in the proximal tubule of the kidney and promotes glucosuria. The results of the Dapagliflozin Effect on Cardiovascular Events-Thrombolysis in Myocardial Infarction (DECLARE-TIMI) 58 trial, which compared the effect of dapagliflozin plus an existing glucose-lowering drug (GLD) treatment with a placebo in patients with type 2 diabetes (T2D) and high cardiovascular (CV) risk, showed that dapagliflozin yielded a primary safety outcome of major adverse CV events [MACEs; nonfatal myocardial infarction (MI), nonfatal stroke, or CV mortality] that was noninferior to the placebo (Wiviott et al., 2019). In contrast, the findings of the Empagliflozin Cardiovascular Outcome Event Trial in Type 2 Diabetes Mellitus Patients (EMPA-REG OUTCOME) and those of the CANaglifozin cardioVascular Assessment Study (CANVAS) trials showed that the SGLT2 inhibitors empagliflozin and canagliflozin resulted in a significantly lower risk of CVD than the placebo (Zinman et al., 2015; Neal et al., 2017). CVD-REAL Nordic and CVD-REAL 2—a part of the Comparative Effectiveness of Cardiovascular Outcomes in New Users of SGLT2 Inhibitors (CVD-REAL) study program that analyzed the effects of SGLT2i treatment on CV in a real-world setting—suggested that SGLT2i was associated with a lower risk of CV events than other GLDs, including dipeptidyl peptidase-4 inhibitors (DPP-4i), metformin, and sulfonylurea (Birkeland et al., 2017; Kosiborod et al., 2017; Kosiborod et al., 2018). However, only a few studies have made a head-to-head comparison of SGLT2i and other GLDs. A study reported that dapagliflozin was associated with a lower risk of MACE and hospitalization for heart failure (HF) than DPP-4i (Birkeland et al., 2017). There is also inconsistency regarding dapagliflozin-specific effects on CVD. Dapagliflozin did not have any effect on the risk of MACE in the DECLARE-TIMI 58 trial, when compared to that by the placebo, while the CVD-REAL Nordic study reported a lower risk of MACE in patients treated with dapagliflozin as compared to those who received DPP-4i (Kosiborod et al., 2018; Wiviott et al., 2019). We studied the risk of MACE dapagliflozin, with DPP-4i as an active comparator, as it showed no significant effect on the risk of MACE in many clinical trials among patients with T2D and is considered to be neutral with regard to CV risk (Lamos et al., 2019).

There is a growing demand for the efficient use of healthcare resources. It has become important to decision makers to choose the best option while considering the clinical and economic benefits of the treatments. Currently, there are no studies on the difference in the medical costs of T2D treatment between patients who used dapagliflozin and those who used DPP-4i, with clinical outcomes. In this study, we aimed to investigate the relative risk of MACE among patients receiving either dapagliflozin or DPP-4i and compare the direct medical cost between the two groups, based on the clinical outcomes from a healthcare provider’s perspective.

## Methods

### Data Sources

In this retrospective cohort study, we used the national health insurance (NHI) claims database maintained by the Health Insurance Review and Assessment (HIRA) services, the single national payer in Korea. All Koreans have a unique identification number, which is mandatory for all administrative purposes, and are covered by the NHI system. The database contains information on patients’ demographics, medical diagnoses (using the 10th International Classification of Diseases codes), and healthcare utilization-related data, such as hospitalizations, physician visits, prescriptions (generic name, prescription date, dose, duration, and route of administration), and in-hospital deaths, of approximately 50 million Korean people (Seong et al., 2011). The entire NHI claims data of patients from September 1, 2014, to June 30, 2018, were used in this analysis. The study protocol was approved by the CHA University Institutional Review Board (protocol ID: 1044308-201812-HR-060-01). Prior research on HF risk has been published previously (Seong et al., 2020).

### Study Population

Patients who fulfilled the following inclusion criteria were enrolled: 1) aged 18–75 years; 2) diagnosed with T2D (ICD-10: E11) between September 01, 2015, and August 31, 2016; and 3) initiated dapagliflozin or DPP-4i treatment during the same period. The cohort entry date was the earliest start date of the study medication therapy. A new user of dapagliflozin or DPP-4i was defined as a person who had not been prescribed dapagliflozin or DPP-4i within 365 days before the cohort entry. Patients were excluded if they: 1) received other SGLT2i and glucagon-like peptide 1 (GLP-1) receptor agonists within 365 days before the cohort entry; 2) had an acute CV event (e.g., hospitalization with a diagnosis of HF, MI, and ischemic stroke) within 8 weeks before the cohort entry (using criteria comparable to those defined in the DECLARE-TIMI 58 trial); 3) had a history of cancer, human immunodeficiency virus infection, or end-stage renal disease at any time during the whole study period; or 4) had been diagnosed with type 1 diabetes or gestational diabetes within 365 days before cohort entry. Patients who were initiated on dapagliflozin and DPP-4i were categorized into the dapagliflozin group and DPP-4i group, respectively.

### Baseline Data

Variables potentially related to the development of CVD were assessed. These baseline variables included age, sex, number of oral hypoglycemic drugs at cohort entry, and microvascular complications of diabetes (nephropathy, neuropathy, and retinopathy). Comorbidities were determined based on the following diagnoses within a year prior to the date of cohort entry: hypertension; dyslipidemia; chronic kidney disease; CVD, including MI, other ischemic heart diseases, ischemic stroke, hemorrhagic stroke, occlusive peripheral arterial disease, coronary revascularization procedures (coronary artery bypass graft, percutaneous coronary intervention), HF, and atrial fibrillation; hypoglycemia; asthma; chronic obstructive pulmonary disease; connective tissue disease; pancreatitis; osteoporosis; alcohol intake; smoking habit; and obesity. For concomitant medication data, patients with one of the following drug prescriptions within 180 days prior to the date of cohort entry were identified: GLDs including metformin, sulfonylurea, thiazolidinediones, alpha-glucosidase inhibitor, meglitinide, and insulin; diuretics, such as loop diuretics, thiazide, aldosterone antagonist, and potassium-sparing diuretics; antihypertensive agents, including calcium channel blocker, angiotensin-converting enzyme inhibitor, angiotensin II receptor blocker, and alpha and beta blockers; digoxin; aspirin; P2Y12 inhibitor; warfarin; non-vitamin K antagonist oral anticoagulant; and lipid-lowering agents such as statin, fibrate, and ezetimibe. In addition, indicators of healthcare utilization, such as visits to the Cardiology department within 30 days; hospitalization within 30 days and/or within 30–365 days; and visits to the emergency department within 365 days prior to the date of cohort entry during the baseline period, were identified.

### Clinical Outcome: MACE

Clinical outcome was defined as the first occurrence of MACE [composite of nonfatal I (admission with ICD-10 code I21), nonfatal stroke (admission with ICD-10 codes I63 and I64), or in-hospital CV death (discharge diagnosis codes I461 and I469)] after the cohort entry date. An on-treatment approach was used for the base analysis. Patients were observed from cohort entry until the incidence of one of the following: MACE, treatment switch or discontinuation, death (except CV death), or end of study period (June 30, 2018). This on-treatment approach was used to minimize the effects of patient emigration, as such patients would be classified under treatment discontinuation, defined as a gap of >30 days (median duration of GLDs prescription) between prescription fill dates. Additionally, intention-to-treat analyses were performed, which assumed that the medications prescribed at the time of cohort entry were used throughout the follow-up period.

### Economic Outcome: Direct Medical Cost

**FIGURE 1:**
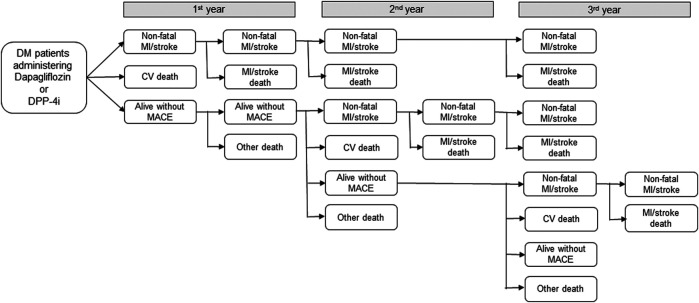
Decision-analytic model for economic analysis. DM: diabetes mellitus, DPP-4i: Dipeptidyl peptidase-4 inhibitors, MI: myocardial infarction, CV: cardiovascular, MACE: major adverse cardiovascular events, Nonfatal MI/stroke: survived after MI/stroke incidence; CV death: CV death without prior MI or stroke; alive without MACE: no onset of MACE; MI/stroke death: death following MI/stroke; and Other death: other than CV death without the diagnosis of MI/stroke.

This analysis was conducted from a healthcare provider’s perspective. For estimating the direct medical costs of the dapagliflozin and DPP-4i groups, the macro-costing approach was used based on HIRA data, containing information of the overall medical service utilization costs, including medication, hospitalization, diagnosis, and treatment per patient by diagnosed health status. A decision-analytic model reflecting the patients’ health status changes during the 3-year period was used (Figure 1). The annual medical costs corresponding to each health status and the health status transition probabilities were applied in the model, and the total direct medical cost for the 3-year period was estimated for 1,000 patient cohorts in both groups. Five different health statuses were included in the model: nonfatal MI/stroke (survived after MI/stroke incidence); CV death (CV death without prior MI or stroke); alive without MACE (no onset of MACE); MI/stroke-related death (death following MI/stroke); and death by other cause (other than CV death without the diagnosis of MI/stroke). Patients under each health status were defined as patients diagnosed with MI, stroke, CV death using ICD-10 codes listed in the clinical outcomes, and death due to other causes (discharge diagnosis codes R95, R96, and R99).

### Propensity Score

Propensity scores (PS) were used to balance the baseline characteristics between the two groups. PS was estimated using a multivariable logistic regression model, with all covariates evaluated as the baseline characteristics in Table 1. We then weighted a Cox proportional-hazard model based on the PS values, in which we used the standardized morbidity ratio (SMR) to compute the risk of MACE in each group. These SMR-weighted analyses used the value one for the treated (dapagliflozin) group and the propensity odds for the untreated (DPP-4i) group as weights (Kurth et al., 2006).

**TABLE 1:** Baseline characteristics of the study population before and after propensity score-weighted analysis.

	Entire population			Propensity score-weighted population		
	Dapagliflozin (N = 23,147)	DPP-4i (N = 237,189)	Standardized difference	Dapagliflozin (N = 23,147)	DPP-4i (N = 237,189)	Standardized difference
Age (years), mean (SD)	52.6 (11.50)	56.9 (10.88)	0.38	52.6 (11.50)	52.7 (3.59)	0.00
Men, %	54.3	59.7	0.11	54.3	54.4	0.00
Microvascular complication of diabetes						
Nephropathy	8.4	6.5	0.07	8.4	8.5	0.00
Neuropathy	12.3	11.2	0.03	12.3	12.3	0.00
Retinopathy	15.7	13.0	0.08	15.7	15.7	0.00
Cardiovascular disease						
Hemorrhagic stroke	0.4	0.6	0.04	0.4	0.4	0.00
Other ischemic heart disease	15.1	11.9	0.09	15.1	15.2	0.00
Ischemic stroke	3.3	4.2	0.05	3.3	3.3	0.00
Myocardial infarction	1.3	1.0	0.03	1.3	1.3	0.00
Heart failure	3.5	2.9	0.04	3.5	3.6	0.00
Occlusive peripheral arterial disease	0.7	0.8	0.01	0.7	0.7	0.00
Coronary artery bypass graft	0.03	0.05	0.01	0.03	0.03	0.00
Percutaneous coronary intervention	1.8	1.5	0.02	1.8	1.8	0.00
Comorbidities^a^						
Obesity	0.4	0.2	0.05	0.4	0.4	0.00
Osteoporosis	6.9	9.2	0.08	6.9	6.9	0.00
Atrial fibrillation	1.6	1.6	0.00	1.6	1.6	0.00
Hypertension	60.5	57.3	0.06	60.5	60.5	0.00
Alcohol intake	4.4	5.6	0.06	4.4	4.4	0.00
Smoking habit	0.2	0.1	0.02	0.2	0.2	0.00
Asthma	12.6	12.2	0.01	12.6	12.6	0.00
Chronic kidney disease	0.7	1.5	0.08	0.7	0.7	0.00
COPD	4.7	5.4	0.03	4.7	4.7	0.00
Connective tissue disease	3.6	3.8	0.01	3.6	3.7	0.00
Pancreatitis	1.6	1.7	0.01	1.6	1.6	0.00
Hypoglycemia	2.3	2.3	0.00	2.3	2.3	0.00
Dyslipidemia	83.6	77.5	0.16	83.6	83.6	0.00
Medication use						
Antidiabetic agents						
Number of drugs	2.0 (0.64)	2.1 (0.64)	0.24	2.0 (0.64)	2.0 (0.20)	0.00
Metformin	83.5	89.1	0.16	83.5	83.5	0.00
Sulfonylurea	38.3	43.1	0.10	38.3	38.5	0.00
Thiazolidinediones	10.8	9.1	0.06	10.8	10.9	0.00
Meglitinide	0.9	0.7	0.01	0.9	0.9	0.00
α-Glucosidase inhibitor	4.2	4.6	0.02	4.2	4.3	0.00
Insulin	19.3	13.6	0.15	19.3	19.2	0.00
Antihypertensive drugs						
Calcium channel blockers	27.5	27.3	0.00	27.5	27.4	0.00
ACEIs	2.5	1.8	0.05	2.5	2.6	0.00
β-Blockers	9.1	7.8	0.05	9.1	9.1	0.00
ARB	45.3	42.2	0.06	45.3	45.2	0.00
α-Blockers	0.7	0.7	0.00	0.7	0.7	0.00
Diuretics						
Thiazides	13.7	13.3	0.01	13.7	13.7	0.00
Aldosterone antagonists	1.9	1.8	0.01	1.9	2.0	0.00
Loop diuretics	3.3	4.0	0.04	3.3	3.4	0.00
Potassium-sparing diuretics	0.06	0.07	0.00	0.03	0.06	0.00
Warfarin	0.6	0.6	0.01	0.6	0.6	0.00
NOACs	0.4	0.6	0.02	0.4	0.4	0.00
Aspirin	21.1	21.6	0.01	21.1	21.2	0.00
P2Y12 inhibitors	11.4	10.1	0.04	11.4	11.5	0.00
Digoxin	0.6	0.8	0.02	0.6	0.6	0.00
Lipid-lowering agents						
Statins	53.4	47.8	0.11	53.4	53.4	0.00
Ezetimibe	6.7	5.0	0.07	6.7	6.7	0.00
Fibrate	6.3	5.0	0.05	6.3	6.3	0.00
Healthcare utilization						
Cardiologist visit	9.1	5.6	0.14	9.1	9.4	0.01
Emergency department visit	7.6	7.7	0.00	7.6	7.6	0.00
Hospitalization^b^	6.5	12.5	0.21	6.5	6.5	0.00
Hospitalization^c^	15.2	15.8	0.02	15.2	15.3	0.00

DPP-4i, Dipeptidyl peptidase 4 inhibitor; SD, Standard deviation; COPD, Chronic obstructive pulmonary disease; ACEI, Angiotensin-converting enzyme inhibitor; ARB, Angiotensin-2 receptor antagonist; NOAC, Novel oral anticoagulant

a Confirmed by diagnosis code (International Classification of Diseases, 10th revision)

b Hospitalization within 30 days prior to index date

c Hospitalization 30–365 days prior to index date

### Statistical Analyses

Standardized differences were used to examine the balance in baseline values before and after PS weighting (Mamdani et al., 2005). Following PS weighting, the Cox proportional-hazards model was used to obtain the hazard ratios (HR) and 95% confidence interval (CI) for MACE in the dapagliflozin and DPP-4i groups. Kaplan–Meier curves were plotted for the cumulative incidence of MACE in each group.

For the economic analysis model, health status transition probabilities were calculated using the HR obtained from the clinical outcome analysis. The 1-year probability of MACE in the DPP-4i group was converted using the MACE incidence rate, and in the dapagliflozin group, it was calculated by multiplying the DPP-4i incidence rate with the aHR. To determine the number of CV deaths per year among MACE cases, the proportion of CV deaths among the total MACE patients in each group was used. The mortality rate due to MI/stroke was calculated based on the data tracked from the MI/stroke occurrence to death or study end date (June 2018) for all study subjects, and it was converted into a 1-year probability for both groups. To estimate the probability of other deaths in the DPP-4i group, patient mortality rate before any MACE event in the DPP-4i group was calculated by following up with the participants from the cohort entry date to death or study end date (June 2018); this was then converted into a 1-year probability. The probability of other deaths in the dapagliflozin group was computed by multiplying the aHR with the incidence rate of the DPP-4i group and converting it into a 1-year probability.

To calculate the medical costs corresponding to each health status, patients were classified as survivors or dead, and the median medical cost per patient per month during the study period was obtained from the cohort entry date to death or study end date (June 2018). CV death was first selected among all dead cases, and the cost for the entire period before CV death was calculated and applied as the CV death cost. For the remaining dead and surviving patients, the cost before and after the occurrence of MI/stroke was calculated and applied. The annual medical cost was calculated as monthly treatment costs × 12 for patients who survived and continued treatment and as monthly treatment costs × 6 for those who died, assuming that they survived for 6 months of the year. In addition, as the database used in this analysis only included the cost of health insurance benefits, the noncoverage rate by disease was applied to estimate the patients’ out-of-pocket medical costs. The noncoverage rate for patients with heart disease as of 2018 (6.3%) was applied for patients with MACE, and the noncoverage rate for all the health insurance beneficiaries (16.6%) was applied for patients without MACE (Choi et al., 2019). All costs were discounted at an annual rate of 5% and presented in US dollars (annual average currency exchange rate for 2018: 1,165 South Korean Won = $1 USD).

The incidence of MACE and HR in the on-treatment analysis was utilized for the base analysis, as the non-adherence in the intention-to-treat analyses could attenuate results towards null, potentially masking the drug effects on outcomes. Intention-to-treat analyses were performed to determine the sensitivity and demonstrate the robustness of the study results. A subgroup analysis was performed to analyze the risk of MACE and the difference in medical costs according to CV comorbidity before the cohort entry date. Patients with underlying CVD were defined based on the following diagnoses during the baseline period: MI, other ischemic heart disease, ischemic stroke, hemorrhagic stroke, occlusive peripheral arterial disease, coronary revascularization procedures, HF, and atrial fibrillation.

SAS version 9.4 and R software version 3.1.2 were used for statistical analyses and the presentation of survival curves.

## Results

**FIGURE 2:**
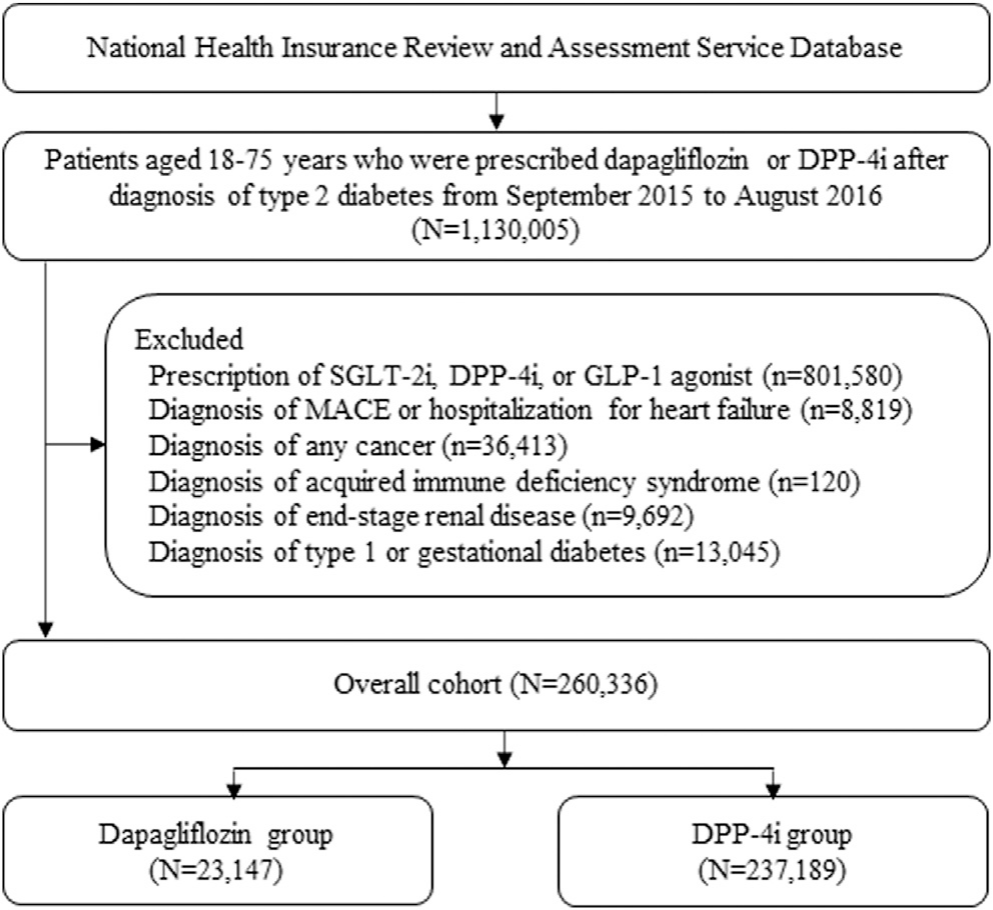
Patient selection flow. DPP-4i: Dipeptidyl peptidase-4 inhibitors, SGLT-2i: Sodium-glucose co-transporter 2 inhibitor, GLP-1: Glucagon-like peptide 1, MACE: Major adverse cardiovascular event.

A total of 260,336 patients were included in the cohort: 23,147 patients were treated with dapagliflozin and 237,189 patients were treated with DPP-4i (Figure 2). Compared with the DPP-4i group, the majority of patients in the dapagliflozin group were young females, treated with a larger number of oral hypoglycemic drugs at cohort entry and diagnosed with dyslipidemia at baseline. The types of oral hypoglycemic drugs at cohort entry varied between the two treatment groups. The dapagliflozin group was more likely to be associated with a history of visits to the cardiologist and less associated with hospitalization within 30 days before cohort entry than the DPP-4i group. After PS weighting, all baseline characteristics of the two groups were well balanced (Table 1).

**FIGURE 3:**
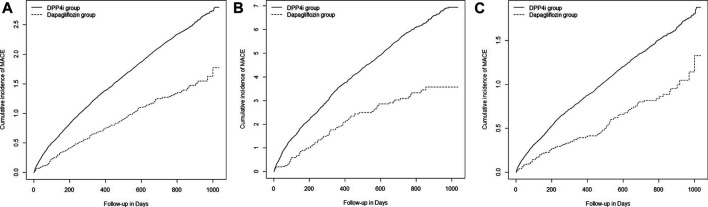
Kaplan–Meier curves for cumulative incidence of MACE in all patients **(A)**, in patients with underlying cardiovascular disease **(B)**, and in patients without underlying cardiovascular disease **(C)**. DPP-4i: dipeptidyl peptidase 4 inhibitor, MACE: major adverse cardiovascular event.

The mean ± standard deviation of the follow-up duration was 1.35 ± 0.96 years. The risk of MACE decreased in the dapagliflozin group compared with that in the DPP-4i group [6.47 vs. 11.33 per 1,000 person-years; adjusted HR (aHR), 0.69 (95% CI 0.57–0.83)]. The aHR of MACE for the dapagliflozin group was 0.64 (95% CI 0.49–0.83) among patients with underlying CVD and 0.76 (95% CI 0.58–0.98) among patients without underlying CVD. In the intention-to-treat analyses, the corresponding aHR was 0.81 (95% CI 0.71–0.92) for the total cohort, 0.79 (95% CI 0.66–0.95) among patients with underlying CVD, and 0.81 (95% CI 0.68–0.97) among patients without underlying CVD (Table 2). Figure 3 shows the Kaplan–Meier curves for the cumulative incidence of MACE.

**TABLE 2:** Risk of MACE: Number of events, person-years, event rates, and hazard ratios of dapagliflozin and DPP-4i groups.

	Dapagliflozin			DPP-4i			cHR	(95% CI)	aHR	(95% CI)
	No. of events	PYs	Incidence (per 1,000 PYs)	No. of events	PYs	Incidence (per 1,000 PYs)				
On-treatment analysis										
Total number of patients	184	28,425	6.47	3,674	324,244	11.33	0.56	(0.48–0.65)	0.69	(0.57–0.83)
Patients with underlying CVDs	89	5,574	15.97	1728	57,399	30.10	0.52	(0.42–0.64)	0.64	(0.49–0.83)
Patients without underlying CVDs	95	22,851	4.16	1946	266,845	7.29	0.56	(0.46–0.69)	0.76	(0.58–0.98)
Intention-to-treat analysis										
Total number of patients	420	53,971	7.78	6,704	543,946	12.32	0.63	(0.57–0.70)	0.81	(0.71–0.92)
Patients with underlying CVDs	208	10,587	19.65	2,934	92,553	31.70	0.62	(0.54–0.72)	0.79	(0.66–0.95)
Patients without underlying CVDs	212	43,384	4.89	3,770	451,393	8.35	0.59	(0.51–0.67)	0.81	(0.68–0.97)

DPP-4i, Dipeptidyl peptidase 4 inhibitor; cHR, Crude hazard ratio; aHR, Adjusted hazard ratio; CI, Confidence interval; PYs, person-years; CVD, Cardiovascular disease.

For cost analysis, model inputs for the clinical outcome parameters according to MACE occurrence and survival/death are presented in Table 3. Patients who died after MI/stroke were found to have the highest annual medical costs (about $7,500), followed by patients who survived a MACE and continued treatment. The annual direct medical cost for diabetic patients with a MACE was $4,672 per patient, which was approximately six times higher than that for diabetic patients without any MACE (Table 4). The estimated direct medical costs over 3 years in 1,000 hypothetical patients treated with dapagliflozin and DPP-4i were $2,461,449 and $2,529,901, respectively, indicating that costs appeared to be lower by $68,452 in the dapagliflozin group as compared to the DPP-4i group. Among patients with underlying CVD, the difference increased to $197,237, six times greater than in patients without underlying CVD ($32,528) (Table 5). The differences in the sensitivity analysis were smaller than those in the base analysis, but the overall trend was similar; for example, the medical cost in the dapagliflozin group was lower than that in the DPP-4i group, and the difference was greater in patients with underlying CVD (Table 5).

**TABLE 3:** Economic analysis model inputs: transition probabilities of dapagliflozin and DPP-4i groups.

Patient groups	Health status	Base analysis		Sensitivity analysis	
	(From → To)	Dapagliflozin group	DPP-4i group	Dapagliflozin group	DPP-4i group
All patients	Alive without MACE → Nonfatal MI/stroke	0.0078	0.0113	0.0099	0.0122
	Alive without MACE → CV death	0.0786	0.0907	0.0786	0.0907
	Nonfatal MI/stroke → MI/stroke death	0.0097	0.0097	0.0097	0.0097
	Alive without MACE → Other death	0.0002	0.0002	0.0002	0.0002
Patients with underlying CVDs	Alive without MACE → Nonfatal MI/stroke	0.0191	0.0297	0.0247	0.0312
	Alive without MACE → CV death	0.0786	0.0907	0.0786	0.0907
	Nonfatal MI/stroke → MI/stroke death	0.0094	0.0094	0.0094	0.0094
	Alive without MACE → Other death	0.0001	0.0004	0.0001	0.0004
Patients without underlying CVDs	Alive without MACE → Nonfatal MI/stroke	0.0055	0.0073	0.0067	0.0083
	Alive without MACE → CV death	0.0786	0.0907	0.0786	0.0907
	Nonfatal MI/stroke → MI/stroke death	0.0100	0.0100	0.0100	0.0100
	Alive without MACE → Other death	0.0002	0.0002	0.0002	0.0002

DPP-4i, Dipeptidyl peptidase 4 inhibitor; MACE, Major adverse cardiovascular events; CVD, Cardiovascular disease; MI, myocardial infarction; CV, Cardiovascular.

**TABLE 4:** Annual direct medical cost by health status per person (USD).

Patient groups	Health status	Annual direct medical cost		
		Health insurance benefit	Out-of-pocket cost	Total
All patients	Nonfatal MI/stroke	4,376.2	295.4	4,671.6
	MI/stroke death	7,017.5	473.7	7,491.2
	CV death	3,229.1	218.0	3,447.1
	Alive without MACE	671.4	111.5	782.8
	Other death	1,308.3	217.2	1,525.4
Patients with underlying CVDs	Nonfatal MI/stroke	4,736.8	319.7	5,056.5
	MI/stroke death	7,194.2	485.6	7,679.8
	CV death	2,993.8	202.1	3,195.8
	Alive without MACE	1,049.8	174.3	1,224.1
	Other death	1,593.3	264.5	1,857.7
Patients without underlying CVDs	Nonfatal MI/stroke	4,128.0	278.6	4,406.6
	MI/stroke death	6,882.0	464.5	7,346.5
	CV death	3,413.1	230.4	3,643.4
	Alive without MACE	615.5	102.2	717.6
	Other death	1,161.7	192.8	1,354.5

CVD: Cardiovascular disease, MACE: major adverse cardiovascular events, MI: myocardial infarction.

**TABLE 5:** Direct medical costs for dapagliflozin and DPP-4i groups over 3 years in hypothetical 1,000 cohort patients (USD).

Patient groups	Treatment	1st year	2nd year	3rd year	3-years total
Base analysis					
All patients	Dapagliflozin group	834,883	820,874	805,691	2,461,449
	DPP-4i group	848,027	844,144	837,729	2,529,901
	Difference between groups	13,144	23,270	32,038	68,452
Patients with underlying CVDs	Dapagliflozin group	1,329,374	1,326,5692	1,319,051	3,974,994
	DPP-4i group	1,367,954	1,393,985	1,410,291	4,172,231
	Difference between groups	38,580	67,416	91,240	197,237
Patients without underlying CVDs	Dapagliflozin group	758,302	739,588	720,499	2,218,390
	DPP-4i group	764,577	750,625	735,717	2,250,918
	Difference between groups	6,275	11,036	15,217	35,528
Sensitivity analysis					
All patients	Dapagliflozin group	843,059	835,677	826,253	2,504,979
	DPP-4i group	851,746	850,812	846,951	2,549,509
	Difference between groups	8,687	15,145	20,698	44,529
Patients with underlying CVDs	Dapagliflozin group	1,350,270	1,363,900	1,370,246	4,084,416
	DPP-4i group	1,373,653	1,404,014	1,423,893	4,201,560
	Difference between groups	23,384	40,113	53,647	117,144
Patients without underlying CVDs	Dapagliflozin	762,746	747,620	731,677	2,242,042
	DPP-4i	768,411	757,498	745,245	2,271,254
	Difference between groups	5,665	9,879	13,568	29,112

DPP-4i, Dipeptidyl peptidase 4 inhibitor; CVD, Cardiovascular disease.

## Discussion

In this large-scale, real-world data-based study, we found that dapagliflozin use is associated with a reduction in the clinical risk of MACE and subsequent reduction in the direct medical costs as well as compared to DPP-4i use.

Two large-scale prospective CV outcome trials (CVOTs)—EMPA-REG OUTCOME and CANVAS—reported that SGLT2i use significantly reduced the primary MACE endpoint compared with the placebo. In the EMPA-REG study, there was a significant reduction in the MACE outcome (nonfatal MI/stroke/CV death) with empagliflozin in patients with T2D and established CVD (Zinman et al., 2015). In CANVAS, canagliflozin significantly reduced MACE (Neal et al., 2017). In contrast to EMPA-REG and CANVAS, the DECLARE-TIMI study with dapagliflozin reported no significant reduction in the MACE outcome. The possible reasons suggested for this discrepancy were the CV benefits of SGLT2i on MACE, such as the heterogeneity in the CVD risk of the study populations. Almost all participants (>99%) in EMPA-REG, 65.6% in CANVAS, and only 40.6% in DECLARE-TIMI had established CVD. In addition, there were differences in the renal function among the three CVOTs, and the better renal function in the DECLARE-TIMI population might be associated with lower CVD events and CV death rates (Hupfeld and Mudaliar, 2019). A meta-analysis of all three SGLT2i CVOTs showed that SGLT2i reduced MACE by 11% (HR 0.89, 95% CI 0.83–0.96), with benefits only in patients with established atherosclerotic CVD (ASCVD) (HR 0.86, 95% CI 0.80–0.93) and no benefits in patients without ASCVD (HR 1.00, 95% CI 0.87–1.16) (Zelniker et al., 2019). Unlike the meta-analysis results, in the current study, dapagliflozin showed a lower risk of MACE, both in T2D patients with and without underlying CVD. Although direct comparisons of these results were difficult due to the differences in subjects and study design, our retrospective cohort study might translate these previous results to a broader and more generalized patient population in a real-world setting.

Several real-world studies compared CV outcomes in SGLT2i versus other GLDs in patients with T2D and a broad CV risk profile. Our study results are consistent with those of the CVD-REAL Nordic study. Persson et al. (2018) showed that dapagliflozin is associated with lower risks of CV events and all-cause mortality than DPP-4i in patients from Denmark, Norway, and Sweden. A US population-based cohort study also reported that dapagliflozin was associated with a decreased incidence of CVD, defined as nonfatal MI or nonfatal stroke, compared with sulfonylureas and DPP-4i. These positive effects of SGLT2i occurred irrespective of the presence of baseline CVD (Dawwas et al., 2019). Our findings are also consistent with those of a network meta-analysis of CVOTs, in which SGLT2i, including dapagliflozin, more effectively reduced MACE than GLP-1 receptor agonists or DPP-4i (Fei et al., 2019). Our study, which was based on a real-world South Korean setting, explains the possible protective effect of dapagliflozin on MACE in T2D patients with and without CVD.

The mechanisms by which SGLT2i exert a cardioprotective effect are not fully understood, even though several possible mechanisms, such as natriuresis and osmotic diuresis, reduction in inflammation, oxidative stress, and arterial stiffness, reduction in blood pressure and body weight, and possible renoprotective effects, have been suggested. These factors could have CV benefits through a range of cardiac effects, including reduction in the left ventricular load, attenuation of cardiac fibrosis and inflammation, and improved myocardial energy production (Verma, 2019). SGLT2i are associated with an increase in the production of alternative fuels for the heart, such as beta-hydroxybutyrate and products of branched-chain amino acid degradation, and their use has been associated with increased cardiac metabolic efficiency (Kaplan et al., 2018). Other possible mechanisms include the inhibition of sodium-hydrogen exchange, increase in erythropoietin levels, and reduction in myocardial ischemia or reperfusion injury (Verma, 2019).

There are several economic studies related to SGLT2i treatment in patients with T2D, evaluating economic outcomes through long-term modeling (Charokopou et al., 2015; Chin et al., 2019; Cai et al., 2019; Nguyen et al., 2018; Neslusan., 2015). SGLT2i, including dapagliflozin, have a relatively short history of clinical use in the market; thus, there is a lack of data on their long-term outcome with respect to efficacy and adverse effects. To evaluate the long-term effectiveness, previous studies have indirectly applied the effects of SGLT2i on diabetes-related attributes such as weight loss, glycated hemoglobin control, and BP control (Charokopou et al., 2015; Cai et al., 2019; Neslusan., 2015). In other studies, the effectiveness derived from clinical trials or observational studies was applied to the initial part of the model only, and assumption-based extrapolation was applied for at least 30 years (Nguyen et al., 2018; Chin et al., 2019). The strength of our study lies in the analysis of clinical outcomes and costs for MACE through real-world data analysis. We used a rather simplified modeling approach to determine the results of the 3-years study using parameter values measured directly from real-world data, unlike long-term modeling approaches that apply numerous assumptions. This has the advantage of minimizing the uncertainties that are inevitable in an economic evaluation model with many assumptions.

Previous economic studies have suggested that SGLT2i are a cost-effective treatment strategy, indicating that the SGLT2i treatment option increases the cost as well as effectiveness (quality-adjusted life year), but the increased cost-effectiveness ratio (ICER) of SGLT2 compared with that of DPP-4i was within a socially acceptable threshold (Charokopou et al., 2015; Neslusan et al., 2015; Nguyen et al., 2018; Cai et al., 2019; Chin et al., 2019). This study addressed the risk of MACE incidence related to dapagliflozin and DPP-4i use, and the clinical risk values were used to estimate the direct medical costs related to MACE occurrence over 3 years. The results of this study showed that dapagliflozin use improved treatment effectiveness and reduced the overall medical cost. The economic benefits probably varied depending on the medical environment, such as medical service fee, and the parameter values and models applied. Unlike previous studies, which showed ICER estimates ($1,512–$76,167), this study noted a lower direct medical cost (approximately $68,452) in the dapagliflozin group compared to the DPP-4i group in a hypothetical 1,000 patient cohort over 3 years. However, it is not appropriate to simply compare this result, presenting the absolute value of reduced direct medical costs, with other studies presenting ICER.

Our study had some limitations. First, we relied on diagnosis codes to determine clinical outcomes. However, previous studies have validated ICD-10 code-based definitions for many important diseases, including MI and ischemic stroke, which were compared using medical record reviews. These reports demonstrated positive predictive values of >70% for MI and 83.4% for ischemic stroke (Park et al., 2000; Kimm et al., 2012). In addition, the overall positive predictive value of the diagnosis was approximately 70% (Park et al., 2003). The mortality data were analyzed based on the diagnosis codes for meddling; thus, they may differ from the actual mortality data. The Korean statistical information service reported that in-hospital CV deaths account for about 75% of the total CV deaths (Korean Statistical Information Service, 2016). However, the incidence rate of omission of CV deaths might not differ between the dapagliflozin and DPP-4i groups. Second, although numerous potential and measurable CV risk factors were accounted for, the results were affected by unmeasured confounders (diabetes duration, body mass index, etc.), which is a universal problem of all observational studies. Third, this study used only MACE as the major clinical outcome and only compared the direct medical costs incurred over 3 years. However, there are several other complications that contribute to the treatment cost of patients with T2D, and the duration of diabetes spans across several decades. For the results of the study to serve as comprehensive pharmacoeconomic evidence for treating diabetes, long-term studies that include other major complications as clinical outcomes are needed. In addition, this study is initiated to help clinicians make practical decisions by pointing out the difference between the two medication treatments already covered by the Korean National Health Insurance. It is not a very sophisticated economic assessment that is carried out to determine whether the drug will be placed on the National Health Insurance Drugs List. Therefore, we used a very simple model, considering that the same health status would result in the same medical cost. The interpretation of the results of this study requires consideration for the initial purpose of this study.

Our study suggests that dapagliflozin use is associated with a lower risk of MACE as compared to DPP-4i use. We also found that the direct medical costs appeared to be lower for the dapagliflozin group than the DPP-4i group. Therefore, dapagliflozin could be a better option than DPP-4i in terms of MACE complications, both clinically and economically.

## Data Availability

The datasets for this article are not publicly available because: it is a national claims database and available upon request to researchers for health-related studies. Requests to access these datasets should be directed to the Health Insurance Review and Assessment services, http://www.xn--cw4bk22a.net/eng/main.do.
